# Application of Sound Waves During the Curing of an Acrylic Resin and Its Composites Based on Short Carbon Fibers and Carbon Nanofibers

**DOI:** 10.3390/ma17215369

**Published:** 2024-11-02

**Authors:** Braian Uribe, Joana Rodrigues, Pedro Costa, Maria C. Paiva

**Affiliations:** Institute for Polymers and Composites (IPC), Campus of Azurém, University of Minho, 4800-058 Guimarães, Portugalmcpaiva@dep.uminho.pt (M.C.P.)

**Keywords:** cymatics, carbon, composite microstructure, dispersion, acoustic waves

## Abstract

Research into particulate polymer composites is of significant interest due to their potential for enhancing material properties, such as strength, thermal stability, and conductivity while maintaining low weight and cost. Among the various techniques for preparing particle-based composites, ultrasonic wave stimulation is one of the principal laboratory-scale methods for enhancing the dispersion of the discontinuous phase. Nevertheless, there is a scarcity of empirical evidence to substantiate the impact of stimulating materials with natural sound frequencies within the acoustic spectrum, ranging from 20 Hz to 20 kHz, during their formation process. The present work investigates the effect of acoustic stimuli with frequencies of 56, 111, and 180 Hz on the properties of an acrylic-based polymer and its discontinuous carbon-based composites. The results indicated that the stimulus frequency affects the cure time of the studied systems, with a notable reduction of 31% and 21% in the cure times of the neat polymer and carbon-nanofiber-based composites, respectively, after applying a frequency of 180 Hz. Additionally, the higher stimulation frequencies reduced porosity in the samples, increased the degree of dispersion of the discontinuous phase, and altered the composite materials’ thermal, optical, and electrical behavior.

## 1. Introduction

Polymer matrix composites containing particle and discontinuous fiber reinforcements have attracted researchers’ attention in material science and engineering due to their combined characteristics of low weight, low cost, stiffness, corrosion resistance, and functional properties [1,2,3]. The definition of a composite material relies on the synergy between phases, usually but not strictly of different natures, in which the resulting material exhibits combined and enhanced properties compared to those presented by each material individually [4,5]. This effect is due to a matrix that serves as a binder to embed a discontinuous phase and a transition zone with different properties, known as the interphase [6,7]. The discontinuous phase, classically known as the reinforcement, can present multiple shapes [8], and dimensional scales [9] can exhibit other exciting properties, such as thermal [10], electrical [11], acoustic [12], magnetic [13], biological properties [14], among others. However, the effectiveness of the composite performance will be significantly influenced by the organization of the filler within the polymer matrix [15]. For this reason, dispersion becomes a decisive factor in composites consisting of particle and short-fiber reinforcements. In many cases, it also represents a significant technical challenge that must be overcome to obtain materials with the minimal possible defects and the desired properties [16,17]. Various methods can be employed to disperse particles in liquid polymer systems, including solvent exchange, surfactants, exfoliation, and ultrasound waves [18]. In solvent exchange, the particles and the polymer are dissolved in a common solvent that will then be eliminated by evaporation, which is effective when the liquid phases are highly compatible [18]; however, due to the presence of toxic solvents, their use is increasingly limited, and more research is necessary for their replacement with greener alternatives [19]. In some cases, surfactants have been an alternative to improve the stability of the particle suspension and its degree of dispersion [20]. However, its incorporation may adversely affect the composites’ thermo-mechanical properties [21].

The exfoliation process increases the filler’s surface area by increasing its degree of dispersion in the polymeric matrix. It is mainly applied to materials that present a lamellar structure, such as graphene or some phyllosilicates [22]. This process requires dispersing agents, solvents or polymeric emulsions and using shear forces or cavitation processes based on ultrasound resulting in the exfoliation of the filler and its subsequent dispersion in the matrix. However, the processes are complex because they must be adaptive to each type of material and may result in structural damage of the filler or its functional properties [23].

A widely used route in the manufacture of particulate composites is based on the use of sonochemistry, in which ultrasound waves between 20 kHz and 10 MHz are applied to break up the agglomerates of particles present in a polymeric suspension, being also useful to produce high concentrations of H· and OH· radicals in water [24,25] and the production of nanomaterials [26]. However, its use is limited to the laboratory scale, and the process can generate side effects such as degradation of the polymer used, changes in particle morphology and costs that hamper its scalability [27,28]. Although ultrasound waves are applied to the manufacturing processes of composites consisting of micro- and nanofillers, in the literature reviewed, there is insufficient evidence of the application of low sound frequencies, i.e., frequencies audible to the human ear, ranging from 20 Hz to 20 kHz, in the structuring of polymers and polymer composites based on discontinuous fillers.

Understanding the interaction between mechanical waves and materials is key to the development of new technological applications and the prediction of material failure phenomena [29]. 

Cymatics is the study of the visual effects produced by sound and vibration [30], an important tool in understanding resonance phenomena. Concerning the effect in fluids, a complete study reported by Sheldrake [31] presents the main experimental factors with the greatest influence on the formation of patterns for frequencies between 50 and 200 Hz.

Several studies related to cymatics focus on the visual analysis of different vibration modes using the Chladni plate and the prediction of natural resonance frequencies of different substrates and geometries by computational simulation techniques [32,33,34,35]. Cymatics has also been successfully applied in removing naturally deposited solid particles on solar panel surfaces, avoiding the decrease in optical properties and thermal transfer of the photovoltaic cells [36]. In another interesting engineering application, cymatics has been used to predict the probability of failure of aircraft engines from the graphical representation of noise based on the patterns formed by the engine in its intact state [30].

Resonance techniques have been applied to structural composite materials to reduce voids during CFRP tube fabrication, improving the final parts’ mechanical properties. [37]. Low-frequency vibrations between 10 and 50 Hz, produced by a laboratory shaker, were utilized to reduce voids and bubbles in composite laminates fabricated by a hand lay-up process [38]. In another study by the same authors [39], frequencies below 8 kHz were applied directly to the surface of a metal mold used in a vacuum-bagging process to fabricate composite laminates. In this study, the rigid mold reached a resonance frequency close to the stimulus frequency, thus achieving an efficiency in void reduction of up to 60%. In recent research, a vibration platform operating between 10 and 5000 Hz was employed as a rigid support to improve the quality of continuous carbon fiber composites manufactured using vacuum-bagging techniques at different autoclave pressure levels. Researchers obtained the best interlaminar shear strength response with a vibration acceleration of 10 g, resulting in a void content of 0.5 vol% without autoclave pressure [40].

In the present work, we propose the application of acoustic waves at natural frequencies on carbon-based discontinuous phases in a liquid acrylic polymer, Elium^®^, to evaluate their influence on particle organization and to correlate these findings with the resulting material properties. We selected stimulus frequencies between 0 and 200 Hz to account for the following aspects: increasing the efficiency in reducing defects in thermosetting polymers during their curing process [41] and considering possible effects of the frequencies produced by industrial equipment, many of which fall within the study range [42]. This last point is of great importance because acoustic frequencies of equipment are not considered processing variables in the manufacturing and synthesis of chemical compounds and materials, which may be a possible cause of process deviations in laboratory and industrial scales.

Thus, this work constitutes a first approach to using low-frequency acoustic waves in the structuring of reactive liquid thermoplastic polymers and their particle-based composites, with potential interest in further designing for functional polymer composites.

## 2. Materials and Methods

### 2.1. Materials

The polymer used was the liquid thermoplastic resin Elium^®^ 188 XO with a viscosity of 100 mPa.s at 25 °C, purchased from ARKEMA (Colombes, France). The initiator was solid benzoyl peroxide (BPO) obtained from Castro Composites (Pontevedra, Spain). A mixing ratio of 3% by weight of BPO, based on the weight of resin, was employed. Chopped carbon fibers (CF) KRECA KCF-100 from Kureha Corporation (Tokyo, Japan), and carbon nanofibers (NF) of the Pyrograph III type from Pyrograf Products Inc. (Cedarville, OH, USA). (former Applied Sciences Inc., Cedarville, OH, USA) were used as fillers. The morphological, mechanical, and electrical properties of the reinforcing fibers, as indicated in the technical datasheets, are presented in Table 1.

### 2.2. Composite Preparation

The composition of each phase was defined by mass for all the systems studied, and then a two-stage mixing and pre-dispersion of these was performed using an IKA (Staufen, Germany) magnetic stirrer. First, 0.7 g of BPO was incorporated in 23 g of resin and mixed for 2 min at 350 rpm. Subsequently, 0.0237 g of dispersed phase, corresponding to 0.1% by mass of liquid matrix, was incorporated into the mixture and stirred for 8 min at 500 rpm. At the end of this stage, the mixtures were poured into 90 mm diameter Petri dishes before acoustic stimulation.

### 2.3. Acoustic Stimulation

**Figure 1:**
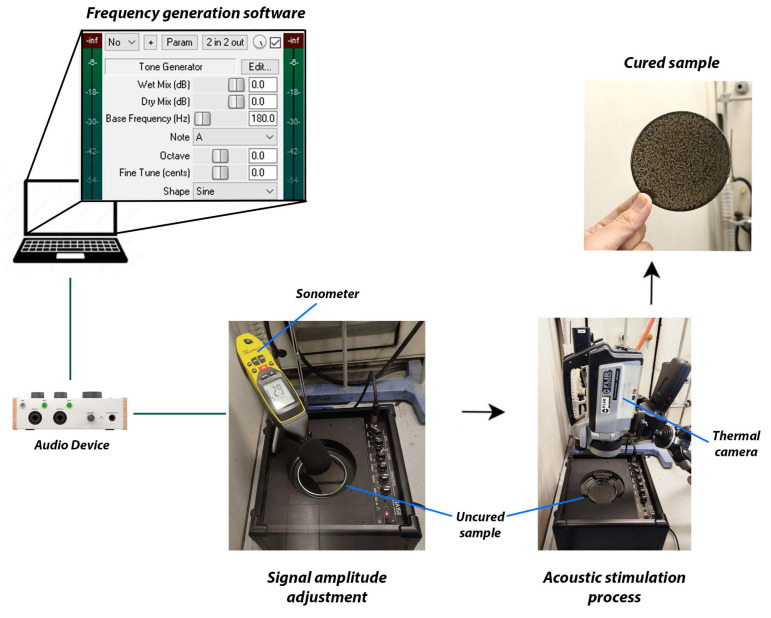
Set-up of the acoustic stimulation experiment.

The acoustic stimulation experiment was performed using a Yamaha GA15II (Hamamatsu, Japan) sound amplifier with a nominal power of 15 W and a frequency response between 50 and 2000 Hz. Table 2 presents the acoustic parameters used in the experiments, measured following the procedure stablished by [43]. The software used as frequency generator was Reaper^®^ v6.81. The acoustic stimulation was maintained during the resin curing process, which was periodically monitored with a thermal camera. The Petri dish was positioned directly over the sound source, as shown in Figure 1, to ensure that the resulting vibration was transferred to the analysis system.

Foam wedges were used between the Petri dish’s walls and the speaker’s internal cavity to avoid lateral movements of the sample during vibration. The stimulus was maintained during the curing time of the material. After 24 h, the cured samples were removed from the mold for further analysis. Thus, the resin (R) and composites with carbon fibers (CFs) and carbon nanofibers (NFs) were studied after curing the resin without and with acoustic stimulation at 0, 56, 111, and 180 Hz, designating the corresponding materials as Rx, CFx, and NFx, where x designates the acoustic frequency applied. Three replicate tests were carried out for each material under each applied frequency.

### 2.4. Characterization Methods

The cure times of the studied systems were obtained from the temperature variations measured using the SC640 ThermaCAM infrared camera from FLIR systems (Wilsonville, OR, USA). The cure time was defined as the time required to reach the material’s exothermic peak.

Differential Scanning Calorimetry (DSC) analysis using a DSC 200 F3 Maia from NETZSCH (Selb, Germany) calorimeter was carried out to find relevant transitions in the polymer and composites. Nitrogen was used as the purge gas at 50 mL·min^− 1^. Thermal properties were recorded after the second heating cycle to erase the thermal history of the polymer. The temperature range was between 20 and 200 °C, and the heating rate was 10 °C·min^− 1^.

Dynamic Mechanical Analysis (DMA) was performed using a Tritec 2000B manufactured by Triton Technologies (Nottinghamshire, UK) in tensile mode, scanning between 0 and 150 °C. The heating rate was 2 °C/min, the oscillating frequency was 1 Hz, and the dynamic force was 1 N. 

Fourier-Transform Infrared Spectroscopy (FTIR) analysis was performed with a 4100 Jasco (Tokyo, Japan) spectrometer in ATR mode with 16 accumulations, 4 cm^−1^ resolution in the wavenumber range of 4000–600 cm^−1^.

The polymers’ and composites’ optical properties were analyzed using a UV–vis spectrophotometer Shimadzu UV-240 1 PC (Kyoto, Japan) in the wavelength range between 200 and 800 nm. Fracture surface observations of the composites were carried out in a NanoSEM FEI Nova 200 scanning electron microscope (OR, United States) using secondary electrons at 10 kV. Before observation, the samples were fractured by bending after being subjected to cryogenic treatment.

A Leica VMHT 80 A microhardness tester (Wetzlar, Germany) was used for the microhardness analysis, where a constant load of 4903 mN was applied for 5 s. Ten indentations were made on each sample. The hardness was calculated using Equation (1), where H is the hardness (GPa), F is the force in (mN), and d1 and d2 correspond to the vertical and horizontal dimensions of the impression in microns.

H = 18544 F/d1 × d2(1)

Electrical volume resistivity measurements were performed using the KEITHLEY SMU 2635B (OH, United States) source and meter to assess the resistivity of parallelepiped-shaped samples. The KickStart Instrument Control Software (version 2.11) facilitated data acquisition and analysis. Sample resistivity was calculated from the slope of the I-V curves obtained by measuring the current intensity while varying the voltage from −10 V to +10 V in 0.5 V increments. These calculations were adjusted to account for the specific geometry of the polymer and composite samples. Electrical resistivity and the corresponding conductivity were calculated, accounting for the measured resistance and the sample geometry.

The specimens were morphologically characterized using a digital microscope (Leica DMS 1000 M, Wetzlar, Germany). The images were acquired in reflection mode at different magnifications.

## 3. Results and Discussion

**Figure 2:**
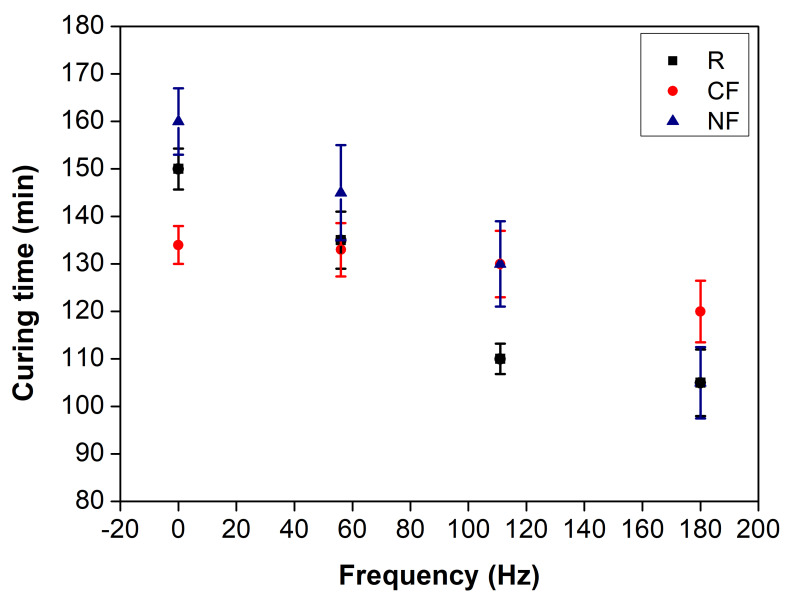
Reaction time of samples versus frequency of the stimulus.

The curing times of the materials tested under different test conditions were measured by recording the temperature variation along the process to observe possible changes induced by the process conditions that could reflect on the exotherm temperatures and reaction times. All the samples studied presented a low exotherm between 28 and 30 °C. Small volumes of material produce thin sample thicknesses, dissipate thermal energy more efficiently, reduce the reaction rate, and increase the cure times, with the heat produced being insignificant in generating pronounced temperature changes [44,45]. In contrast, the variation in the frequency conditions induced important changes in the cure times, as shown in Figure 2, and the cure time of the evaluated systems decreased as the frequency increased. Higher frequencies facilitate enhanced molecular interactions within the resin, resulting in more efficient dispersion of the reagents and an accelerated overall curing reaction. Figure 2 also shows that the reduction in cure time varied for the resins without acoustic stimulus (0 Hz) and higher stimulus (180 Hz) conditions. It was higher for the base resin, showing a decrease of 31%, followed by the NF- and CF-based composites with reductions of 21% and 13%, respectively. This indicates that the discontinuous phase in the composites acts as a dissipative barrier to the mechanical force produced by the acoustic stimulus [46], limiting the fluid motion and reducing the molecular interactions that benefit the polymer cure. It is also inferred from Figure 2 that lower cure times were obtained for the resin and carbon-nanofiber-based composite conditions. Additionally, it is observed that with the application of a frequency of 56 Hz, reductions of less than 15% in the cure time of the systems studied were obtained.

**Figure 3:**
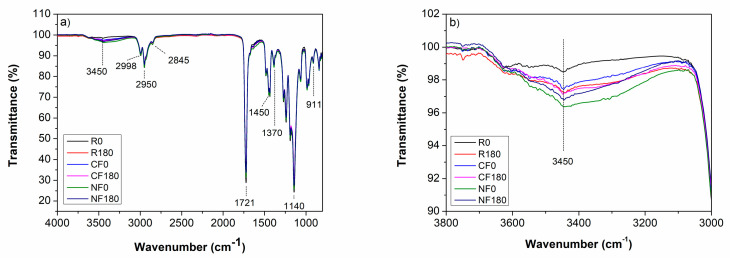
(**a**) FTIR spectra of polymers and composites with and without acoustic stimulus; (**b**) magnification of the region of interest containing the functional group OH-.

The resins and composites were analyzed by FTIR-ATR to observe possible chemical variations in the functional groups that the acoustic stimulus might induce. The spectra presented in Figure 3 are typical of acrylate polymers [47], presenting the -CH_3_ and -CH_2_ stretching vibrations in the region of 3000–2800 cm^−1^, the stretching vibrations of the ester carbonyl groups (-COOR) at 1721 cm^−1^, and the symmetric bending vibration modes of -CH_2_ and -CH_3_ at 1450 and 1370 cm^−1^, respectively. The fingerprint vibrations of PMMA were observed at 911 and 1370 cm^−1^, related to symmetric and asymmetric deformations of the -O-CH_3_ group [48] and bending of CH_3_, respectively. The peaks near 1140 cm^−1^ are related to the stretching vibrations of the C-O-C bond. Finally, the wide band centered near 3450 cm^−1^ is due to hydrogen-bonded O-H stretching vibrations that may originate from moisture absorption. The spectra show a slight decrease in transmission (increase in absorption) for the stimulated condition and after the incorporation of fillers in the polymer, as observed in Figure 3b. This could be associated with moisture at the surface of the dispersed phase, with the nanofibers inducing a higher effect due to their larger surface area [49]. Due to the absence of polar groups in the non-polar PMMA polymer, the probability of hydrogen bonding at the matrix–filler interface is negligible. So, the band observed by FTIR may be assigned to -OH originating from water contamination.

**Figure 4:**
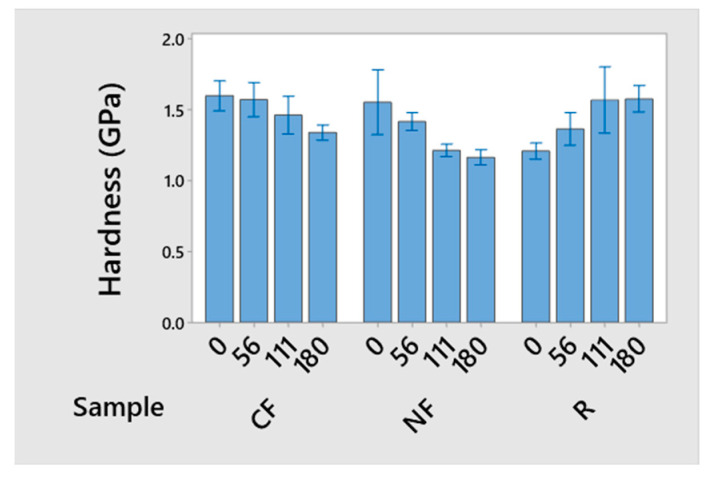
Hardness results for polymers and composites with and without application of acoustic stimulus.

The resins and composites were subjected to microhardness measurements, and the results obtained for the mean hardness and corresponding standard deviations are presented in Figure 4. Additionally, average values for each condition, without post-curing treatments, were plotted to provide a clearer view of the obtained behavior. Figure 4 shows the hardness variation with the frequencies applied during polymerization for R and its composites after curing at room temperature. It was observed that, without applying external stimuli, the composites exhibited average hardness values higher than those of the resin. Specifically, the unstimulated composites based on CF and NF showed hardness values nearly 20% higher than the neat resin. With the application of acoustic stimuli, a decreasing trend is observed in the average hardness values with increasing stimulus frequency for the composites. At the same time, the resin presents an increase in hardness. In all the conditions studied, the hardness of the CF composites was higher than that of the NF composites.

**Figure 5:**
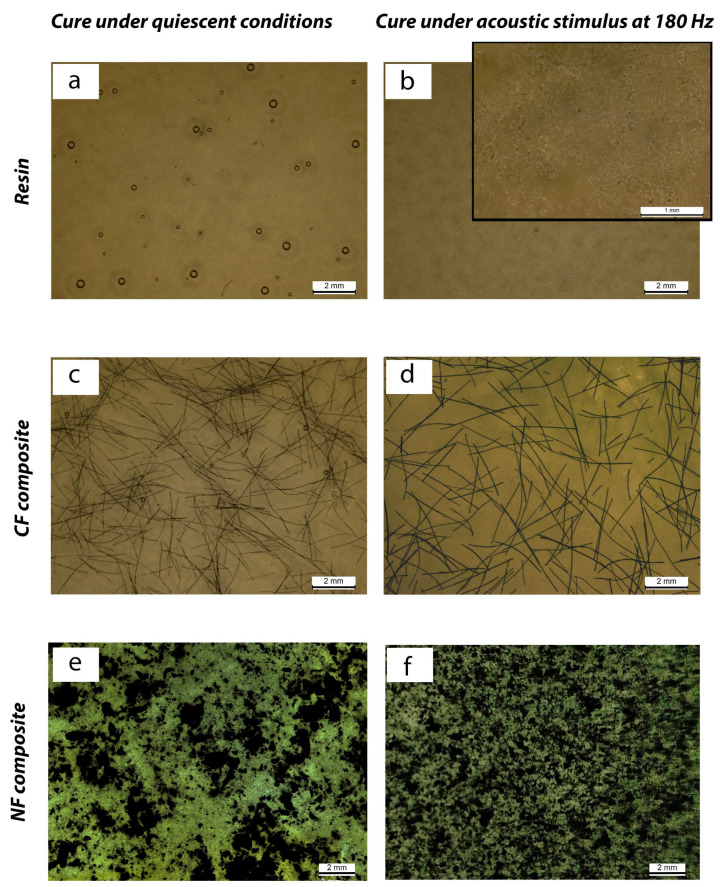
Micrographs of resin (**a**–**b**), CF (**c**–**d**) and NF (**e**–**f**) composites cured without acoustic stimuli and with application of 180 Hz.

The optical micrographs presented in Figure 5 depict a considerable number of microvoids (air bubbles) inside the resin cured without acoustic stimulation, as shown in Figure 5a. At the same time, smaller voids are observed when the cure is carried out under a higher acoustic frequency. The neat resin cured under quasi-quiescent conditions shows air bubbles trapped in the polymer, with diameters below 500 µm, formed during slow stirring of the curing initiator, despite the low viscosity of the resin, the low stirring speed and the low thickness of the sample prepared. The wave’s energy is related to the square of its amplitude, which, for this experiment, was maintained constant; however, the wave propagation rate was changed according to the frequency increase [38]. The reduction in the void size for the stimulated conditions is associated with different combined mechanisms. Viscosity reduction due to vibration is one of the possible mechanisms, as described in [38]. Vibration can create alternating low- and high-pressure zones within the system. In low-pressure areas, bubbles tend to expand, while in high-pressure areas, they compress. This can facilitate the release of bubbles trapped inside the liquid, allowing them to rise to the surface [50]. The bubble reduction due to vibration stimulus also relies on different mechanisms like bubble dissolution followed by coalescence by the Thomson–Flundlich effect and diffusion and growth, which could be amplified if the stimulation frequency is close to the natural resonance frequency of the system [39].

The observed void sizes after stimulation presented diameters below 25 µm, which correlates with the increase in hardness observed [51] and may also affect the material’s mechanical properties. Furthermore, considering that the maximum indentation area was approximately 0.06 mm², voids with a diameter of a few hundred microns will primarily affect the hardness measurements. Additionally, the presence of a wavy surface, observed in Figure 5b, presenting a heterogeneous structure (insert in Figure 5b), suggests a change in the microstructure of the polymer and could explain part of the physical and mechanical properties obtained for the resin samples.

The preparation of CF and NF composites presented different challenges: the optical micrographs of the composites show the presence of fewer and smaller voids compared to the neat resin, when the resin is cured under quiescent conditions. Curing under the acoustic stimuli enhances the dispersion of the carbon fibers and nanofibers, apparently reducing the size and occurrence of voids.

Figure 5c shows the presence of air bubbles in the CF composites prepared under quasi-quiescent conditions; however, they are fewer in number and smaller than those observed in the neat polymer. This indicates that the presence of carbon fibers during the premixing process interferes with the stability of the air bubbles, eliminating the larger ones [52]. However, good fiber distribution was attained with small fiber agglomerates, which are further dispersed when the composite is subjected to a 180 Hz acoustic stimulus (Figure 5d). Under the image acquisition conditions, no air bubbles could be observed by optical microscopy in the composites processed with acoustic stimulation.

The carbon nanofibers, with diameters at least two orders of magnitude smaller than the fibers, cannot be observed individually by optical microscopy. However, the agglomerates and their distribution inside the polymer are depicted in Figure 5d,e. Considering that the optical microscopy images of the composites prepared without and with acoustic stimulus were acquired under the same conditions, it is evident that the composites prepared without acoustic stimulus display larger nanofiber agglomerates and a lower fraction of dispersed nanofibers compared to those treated with acoustic stimulus. Smaller agglomerates are observed in the latter, and the dispersed nanofiber phase absorbs more light, resulting in darker images.

**Figure 6:**
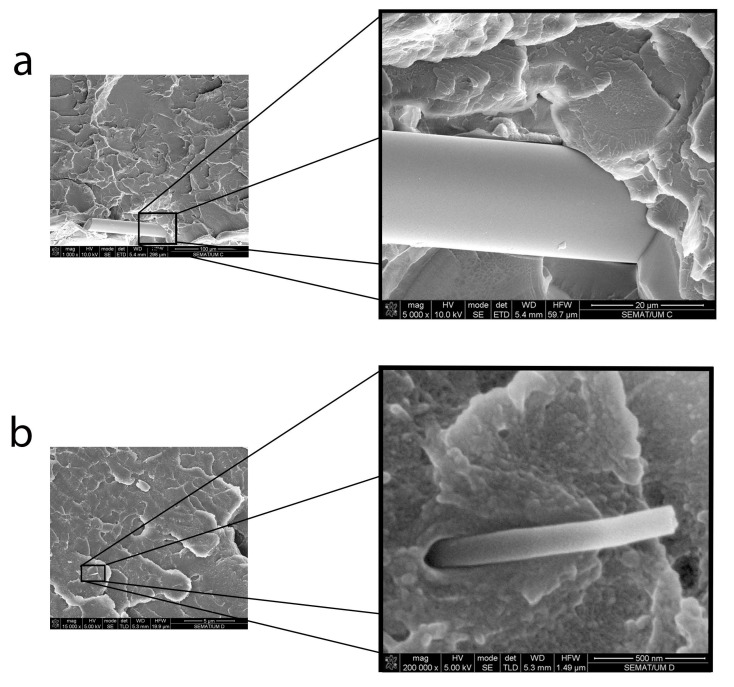
SEM micrographs illustrating the weak interfacial bonding in (**a**) CF and (**b**) NF composites.

More effective dispersion of the fibers or nanofibers will lead to forming a larger area of fiber/polymer interfaces, which may be potential sites for stress concentrations and defect generation, especially when the interface formed is weak, thus leading to interfacial debonding. The poor fiber–matrix interface is evident for both CF and NF composites, as displayed in Figure 6a and Figure 6b, respectively, which facilitates failure during the indentation tests [9]. Thus, while the greater hardness of the composites relative to the neat resin is expected, associated with the presence of high-modulus carbon fibers or nanofibers, the decrease observed with the application of the acoustic stimuli may originate from interfacial effects.

Differential scanning calorimetry was performed to estimate the possible effects of the acoustic treatment on the thermal transitions of the polymer and composites treated in different conditions. The acrylic resin Elium^®^ is an amorphous polymer. Therefore, its glass transition temperature (Tg) is a relevant thermal transition that may be affected by the presence of fibers, nanofibers, or physical actions applied during the cure of the resin [46]. The solid-to-liquid transition of Elium^®^ is assigned to the increase in free volume resulting from the thermal energy gain of the polymer chains with increasing temperature, molecular vibration, and distance between the polymer chains [53,54]. The glass transition temperatures of the polymer and composites are displayed in Table 3.

The resin showed similar Tg values under both base and stimulated conditions at 180 Hz, with readings of 79.8 °C and 80.3 °C, respectively. This similarity was anticipated, as both samples were composed of a single phase. The presence of pores in the sample could have slightly reduced the stiffness of the polymer and increased its free volume, also increasing the mobility of the polymer chains at lower temperatures, which was observed as a lower Tg [55,56]. 

The CF and NF showed an increase in Tg for the composites prepared with acoustic stimulation, which may be related to the increased dispersion of the fibers and nanofibers. A better filler dispersion in the polymer will enhance the establishment of a lattice that limits the movement of the polymer chains, thus shifting the Tg to a higher value.

**Figure 7:**
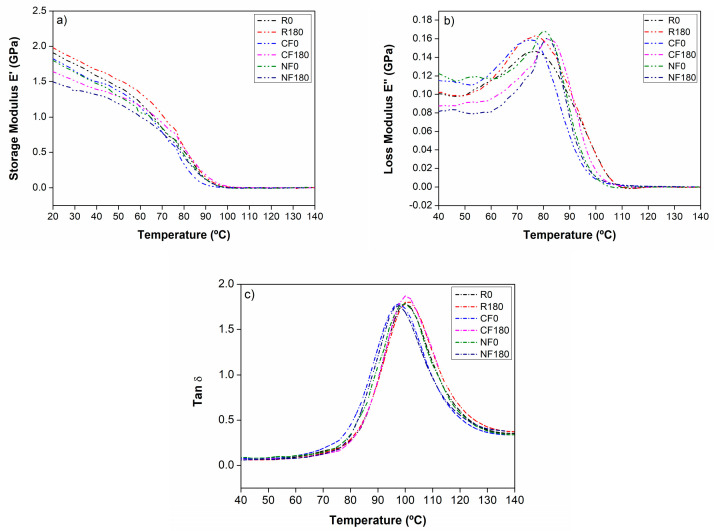
(**a**) Storage modulus; (**b**) loss modulus; and (**c**) Tan d curves for resin and composites.

The storage modulus (E′), loss modulus (E″), and Tan δ curves presented in Figure 7 were analyzed to evaluate changes in the materials with and without acoustic stimulus. When observing the elastic modulus of the systems (Figure 7a), it is evident that in the glassy region, the neat resin presents values close to those under the 180 Hz-stimulated condition. The increase in stiffness can be attributed to the reduced voids of the treated polymer.

The stimulated composites presented reductions in the storage modulus at room temperature, correlating with the hardness values measured at 25 °C. Although hardness and storage modulus have different properties, both depend on the molecular structure, filler–polymer interfaces, and defects. Thus, the hierarchy of values obtained from the hardness analysis—where the resin shows the highest stiffness, and the NF-based composites exhibit the lowest mechanical response under the 180 Hz condition—was corroborated by the DMA results that present a similar trend.

Better dispersion of the fillers and reduced defects in the material are usually associated with improved mechanical performance of composites. However, the mechanical detriment observed in the CF and NF samples may be mainly due to the poor compatibility between phases, as observed in the SEM images, as shown in Figure 6. This incompatibility may lead to stress concentrations at the fiber–matrix interface, which increase the number of defects and contribute to the reduction in mechanical properties, observed as a reduction in the E’. The most significant reduction in storage modulus was observed for the NF composite, which is expected since a smaller fiber size is associated with a larger surface area of the filler, and thus a large increase in interface within the composite, significantly increasing the number of interfacial micro-defects. Another detrimental factor is the moisture present in the reinforcing phase, as corroborated by FTIR analysis in Figure 3, with the water molecules acting as a plasticizer for the polymer, which may contribute to the reduction in E’ values at higher levels of dispersion in comparison to those for the unstimulated conditions and polymer samples.

As the curve declines and the glass-to-rubber transition region is observed, the values for all samples converge. Around 100 °C, the elastic modulus drops completely, showing no evidence of different crosslink densities in the polymer or any strengthening effect of the dispersed phase at high temperatures. The loss modulus, E″ in Figure 7b, shows Tg values close to those measured by DSC, ranging from 74 to 82 °C for all materials. Generally, the composites subjected to acoustic waves showed higher Tg values. The Tg values obtained at the tan δ peak occur at a higher temperature than those obtained at the E″ peak; in the present case, an increase of approximately 15 °C was observed. The differences between polymer and composites with and without acoustic treatment are slight, with the Tg values ranging from 97 to 100 °C with similar curve shapes. Thus, it may be inferred that the viscous dissipation in these materials is mainly determined by the polymer, which is reasonable considering the small fraction of CF or NF in the composites.

When comparing untreated conditions, the Tg values, indicated by the E″ peak, were slightly lower for the composites than for the base resin. This behavior could correlate with the OH- bands found by FTIR, suggesting a plasticization effect of the resin due to water uptake following the incorporation of the fillers [57]. 

Due to the high matrix content in each composite system, a resin-dominant behavior was expected, with minimal variations in terms of viscoelasticity. The Tan δ curves, shown in Figure 7c, corroborated this effect, which showed similar peak widths and heights across the tested sample groups. The shifts in temperature under different conditions were attributed to the restriction of polymer chain motions, indicating higher Tg values for the CF composite and neat resin samples stimulated at 180 Hz.

**Figure 8:**
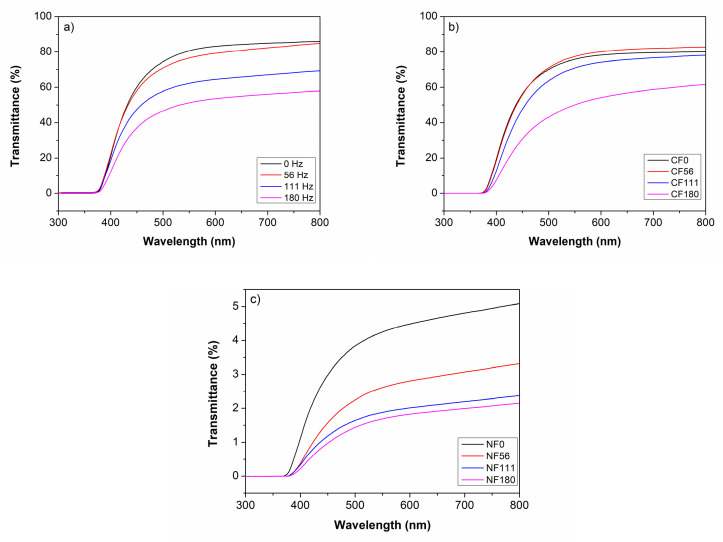
UV–vis curves for (**a**) polymers; (**b**) CF composites; and (**c**) NF composites.

UV–vis spectrophotometry was performed for polymers and composites to analyze microstructural changes based on the interaction with UV–visible light transmitted across the solid samples, as presented in Figure 8a–c. The neat resin and the composites, both with and without acoustic stimulus, show total absorption of light below 375 nm in the ultraviolet region of the electromagnetic spectrum.

An increase in transmittance is observed for all test conditions above 375 nm and up to 800 nm wavelength (primarily the visible region). Resin samples show the highest light transmittance, followed by CF and NF composites. A clear trend for a transmittance reduction with increasing acoustic stimulus frequency is observed for all compositions. For comparison, Table 4 presents the transmittance values at 550 nm (green region) for the polymers and composites with and without applied acoustic stimulus during their curing process.

There was a considerable decrease in transmittance at the 550 nm wavelength for the untreated acoustic stimulus and that treated with 180 Hz, namely, 37, 34, and 59% for the neat resin, CF, and NF composite systems, respectively. The CF-based composites exhibit a behavior like that of the resin due to the low fiber fraction. Consequently, given their size, the number of fibers present in the composite is significantly lower than the number of nanofibers, leading to distinct optical effects. In the case of nanofibers, light scattering is expected to be considerable, with the extent of scattering increasing proportionally to the dispersion quality of the nanofibers. This results in the material becoming opaque [58].

Consequently, optical microscopy confirmed that more excellent dispersion of agglomerates in the NF composites resulted in increased absorption of visible light and a darkening of the material. The NF-based composites showed the most significant reduction in transmittance, supporting the observation of improved dispersion of the discontinuous phase. This reduction in transmittance is attributed to changes in the microstructure of the polymer, as also observed through optical microscopy. Additionally, the stimulated polymer exhibited a multiphase structure that significantly affected its interaction with light. The various phases present in the polymer, stimulated at 180 Hz, likely acted as light-scattering microdots, resulting in increased optical paths and light absorption [59].

The electrical resistivity measurements of the materials analyzed are presented in the Supplementary Materials, Figure S1.

The resistivity values do not differ significantly between the systems under stimulated and non-stimulated conditions. They remain similar and within the same order of magnitude (10 × 10^8^ to 10 × 10^9^ Ohm.cm). These results suggest that the polymer’s inherent resistivity primarily governs the electrical resistivity. In the case of CF and NF composites, the conductive filler fraction was too low to significantly impact the overall electrical responses [60,61].

## 4. Discussion

This research, which is at an initial and exploratory phase, demonstrated, using the employed methodological elements, the effect of acoustic frequencies on the properties of a polymer and two composite systems based on short carbon fibers and carbon nanofibers. This research tested the hypothesis that introducing acoustic waves at frequencies of 56, 111, and 180 Hz during the polymerization process could cause variations in the structure and properties of the polymer and its composites with CF and NF. These results were then compared to systems not subjected to acoustic stimulation. The observed effects are reported, illustrating that applying low-frequency acoustic stimuli during the production of composites with low reinforcement loading affects the filler distribution and the composite properties.

As the wave increased its propagation speed in the liquid medium, there was greater efficiency in the molecular interactions of the polymer matrix, enhancing the reaction efficiency. This effect was observed as shorter times in the cure kinetics of the stimulated systems compared to the reference systems. This research also corroborated the positive effect of acoustic vibration in reducing the size of voids and bubbles present in reactive liquid polymers during their polymerization, as previously indicated by other authors [38,39,40,41]. However, the present study obtained the best results with stimulus frequencies higher than those reported [38,39]. The difference lies in the fact that in this study, the wave amplitude was kept constant throughout the experiment, regardless of the frequency used. Moreover, the experiments presented here were conducted at constant temperatures, without the application of vacuum or high pressure. For this reason, we consider that the results obtained are mostly due to the variation in stimulus frequency and not any additional energy applied to the system or the effect of another external source.

In this research, two-component polymer systems were used, along with carbon-based fillers, without incorporating dispersing agents, degassing agents, or compatibilizers that could alter the physicochemical nature of the phases that constitute the studied systems. Furthermore, a low filler fraction was utilized to facilitate its movement in the liquid medium due to the action of the acoustic waves. For future works, other polymer systems, the effect of polymer viscosity, filler density, shape factor, chemical nature, and compatibility of the filler with the continuous phase should be studied to estimate their influence upon filler distribution, dispersion and composite properties. These suggested approaches will allow for a broader understanding of the structuring process of polymers and composites using non-ultrasonic acoustic frequencies.

This work presents results that could be a starting point for experimental developments in industrial environments. Based on these findings, a research pathway could be opened, incorporating the analysis of different acoustic frequencies produced by equipment and machinery during various polymer and composite synthesis or manufacturing processes.

With this research, we propose the possibility of observing matter in constant vibration, how this effect affects its structure, and what we perceive and understand from it.

## 5. Conclusions

This study examined the effect of the application of 56, 111, and 180 Hz sound frequencies during the curing process of an acrylic-based polymer and its composites based on carbon fibers and nanofibers and their resulting physical–mechanical properties. The principal conclusions are as follows:

The increase in sound frequencies resulted in shorter curing times for polymers, CF- and NF-based composites samples, which presented reduction times of 31%, 21%, and 13%, respectively.

Microhardness exhibited a direct correlation with increasing stimulus frequency in the polymeric system. Conversely, with the increased applied frequencies, the degree of dispersion resulted in lower hardness and storage modulus values for the composites. These results corroborate the low fiber–polymer interfacial bonding, which may lead to more extreme effects when the reinforcing phase is more homogeneously dispersed. FTIR corroborates that residual water may induce a plasticizing effect, also observed as a reduction in hardness and storage modulus.

An increase in the stimulus frequency was observed to result in a reduction in the size and number of the air bubbles trapped in the polymer across all systems under study. Additionally, microscopic examination revealed that higher frequencies are associated with a greater degree of dispersion of the discontinuous phase within the composite.

The polymer and composites exhibited relatively similar values of the glass transition temperature, with a tendency to increase after the application of acoustic stimulus.

Optical properties decreased with the increasing acoustic stimulation frequency for all systems, indicating a higher dispersion of fillers in the composite samples.

For the nanofiber-based system, increasing the frequency led to an enhancement in filler dispersion. The electrical properties were matrix-dominated given the minimal amount of conductive phase present.

## Figures and Tables

**Table 1:** Nominal properties of the reinforcing fibers and nanofibers.

Property	CF	NF
Fiber Diameter	12.5–18 (μm)	60–150 (nm)
Density (g/cm^3^)	1.65	1.8
Tensile Strength (GPa)	0.59	2.7–7.0
Tensile Modulus (GPa)	30–37	400–600
Electrical Resistivity (μΩ-cm)	15,000	55–1000
Fiber Average Length (μm)	~3000	>100

**Table 2:** Parameters for the acoustic stimulation process.

Acoustic Parameters	
Frequencies (Hz)	0, 56, 111, 180
Wave Type	Sine
Sound Pressure Level (dB)	75.0
Applied Sound Power (W)	5.0

**Table 3:** Glass transition temperatures of resin and composites under 0 and 180 Hz of acoustic stimulus.

Sample	Frequency (Hz)	Tg (°C)
R	0	79.8
	180	80.3
CF	0	73.0
	180	83.9
NF	0	78.4
	180	82.3

**Table 4:** Optical transmittance at a wavelength (550 nm) for polymers and composites.

Sample	Transmittance (%)
R0	80.6
R56	76.5
R111	62.2
R180	51.2
CF0	75.8
CF56	76.1
CF111	70.8
CF180	50.1
NF0	4.2
NF56	2.6
NF111	1.9
NF180	1.7

## Data Availability

The original contributions presented in the study are included in the article/Supplementary Materials, further inquiries can be directed to the corresponding author.

## Supplementary Materials

The following supporting information can be downloaded at: https://www.mdpi.com/article/10.3390/ma17215369/s1. Figure S1. Electrical resistivity of polymers and composites at 0 and 180 Hz of acoustic stimulus.
