# Supplementary material for: Application of Sound Waves During the Curing of an Acrylic Resin and Its Composites Based on Short Carbon Fibers and Carbon Nanofibers
Source: Materials (Basel). 2024 Nov 2;17(21):5369. doi: 10.3390/ma17215369 (PMC11547792; doi:10.3390/ma17215369)
Supplement: Supplementary file 1 [file materials-17-05369-s001.zip › materials-3266056-supplementary.pdf]

Application of sound waves during the curing of an acrylic resin and its composites based on short carbon fibers and carbon nanofibers

Braian Uribe[1]*, Joana Rodrigues[1], Pedro Costa[1], and Maria C. Paiva[1]

[1] Institute for Polymers and Composites (IPC), University of Minho, Campus of Azurém, 4800-058 Guimarães, Portugal.
* Correspondence: brianes@dep.uminho.pt

SUPPLEMENTARY INFORMATION

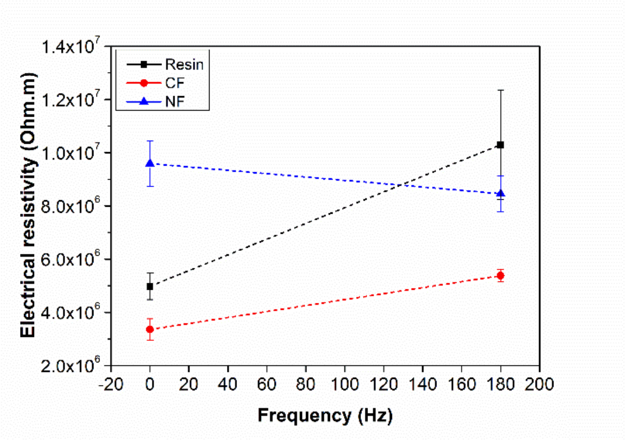

Figure S1. Electrical resistivity of polymers and composites at 0 and 180 Hz of acoustic stimulus.
